# Supplementary material for: Stacking Model‐Based Classifiers for Dealing With Multiple Sets of Noisy Labels
Source: Biom J. 2025 Mar 12;67(2):e70042. doi: 10.1002/bimj.70042 (PMC11898607; doi:10.1002/bimj.70042)
Supplement: Supplementary file 1 — Supporting Information [file BIMJ-67-e70042-s001.zip › density-based-ensemble-model-main/competitors_real_data/netlab-master/netlab-master/help/demgp.htm]

Netlab Reference Manual demgp


# demgp

## Purpose

Demonstrate simple regression using a Gaussian Process.

## Synopsis

```
demgp
```

## Description

The problem consists of one input variable `x` and one target variable
`t`. The values in `x` are chosen in two separated clusters and the
target data is generated by computing `sin(2*pi*x)` and adding Gaussian
noise. Two Gaussian Processes, each with different covariance functions
are trained by optimising the hyperparameters
using the scaled conjugate gradient algorithm. The final predictions are
plotted together with 2 standard deviation error bars.

## See Also

`gp`, `gperr`, `gpfwd`, `gpgrad`, `gpinit`, `scg`

---

**Pages:**
Index


---

Copyright (c) Ian T Nabney (1996-9)
